# Floating Mass Transducer als Mikrofon – ein Pilotversuch

**DOI:** 10.1007/s00106-025-01596-2

**Published:** 2025-04-16

**Authors:** Stefan Kaulitz, Carolina Köstler, Kristen Rak, Rudolf Hagen, Stephan Hackenberg, Mario Cebulla

**Affiliations:** 1 Klinik und Poliklinik für Hals-Nasen-Ohrenheilkunde, Kopf- und Hals-Chirurgie, Universitätsklinikum Würzburg, Würzburg, Deutschland; 2 HNO GROß UND KLEIN, Praxis Dr. med. Stefan Kaulitz, Friedrich-Stein-Straße 9, 97421 Schweinfurt, Deutschland

**Keywords:** Mittelohr, Prothesen und Implantate, Mittelohrimplantat, Implantierbare Neurostimulatoren, Cochleaimplantat, Middle ear, Prostheses and implants, Middle ear implant, Implantable neurostimulators, Cochlear implant

## Abstract

**Hintergrund:**

Die Studie untersucht die inverse Nutzung des Floating Mass Transducer (FMT) der Vibrant Soundbridge® (Fa. MED-EL, Innsbruck, Österreich) als Mikrofon in einem Modellversuch. Sollte dies anwendbar sein, ergäben sich interessante Anwendungsmöglichkeiten z. B. als Mikrofon eines vollimplantierbaren Cochleaimplantats.

**Material und Methoden:**

Durch experimentelle Messungen an einem Trommelfell-Gehörgangs-Modell wurden die akustischen Eigenschaften des FMT bei Verwendung als Mikrofon, einschließlich Frequenzantwort und Empfindlichkeit, analysiert. Der FMT aus dem Direct-Drive-Simulations-Set war hierfür auf dem künstlichen Trommelfell angekoppelt.

**Ergebnisse:**

Die Ergebnisse zeigen, dass der FMT über den gesamten untersuchten Frequenzbereich eine brauchbare Signal-Rausch-Leistung aufweist, allerdings mit einer nichtlinearen Frequenzcharakteristik. Die höchste Empfindlichkeit zeigte sich zwischen 1500 und 2000 Hz.

**Schlussfolgerung:**

Die Studie legt nahe, dass ein auf Mikrofoneigenschaften optimierter FMT als Mikrofon im Mittelohr eingesetzt werden könnte, was neue Möglichkeiten für die Entwicklung vollständig implantierbarer Hörsysteme eröffnen würde. Weitere Untersuchungen, insbesondere Messungen am Felsenbein, sind erforderlich, um die Eignung des FMT als Mittelohrmikrofon genauer zu bestimmen.

## Abstract (en)

**Background:**

This study investigates the inverse use of the Vibrant Soundbridge® Floating Mass Transducer (FMT; MED-EL, Innsbruck, Austria) as a microphone in a pilot test. Should this be applicable, it would open up interesting application possibilities, e.g., as a microphone for a fully implantable cochlear implant.

**Materials and methods:**

Experimental measurements on an ear canal–eardrum model were used to analyze the acoustic properties of the FMT when used as a microphone, including frequency response and sensitivity. The FMT from the Direct Drive Simulation Set was coupled to the artificial eardrum for this purpose.

**Results:**

The results show that the FMT has a usable signal-to-noise performance over the entire frequency range investigated, albeit with a non-linear frequency characteristic. The highest sensitivity was found between 1500 and 2000 Hz.

**Conclusion:**

The study suggests that an FMT optimized for microphone properties could be used as a microphone in the middle ear, which would open up new possibilities for the development of fully implantable hearing systems. Further investigations, in particular measurements on the petrous bone, are required to determine the suitability of the FMT as a middle ear microphone more precisely.

Cochleaimplantate stellen seit vielen Jahren den Standard in der Hörrehabilitation von Taubheit und hochgradiger Schwerhörigkeit dar. Bis heute sind alle auf dem Markt zugelassenen Systeme teilimplantierbar. Vollimplantierbare Systeme hätten attraktive Vorteile. Für die Entwicklung solcher Systeme ist ein implantierbares Mikrofon essenziell.

## Mittelohrmikrofone

Der Floating Mass Transducer (FMT) ist ein aktiver Masseschwinger des teilimplantierbaren aktiven Mittelohrimplantats (AMEI) Vibrant Soundbridge® (Fa. MED-EL, Innsbruck, Österreich). Er besteht aus einem ca. 2 mm großen Gehäuse, in dem sich ein Magnet und eine Spule befinden. Der FMT wird an die Gehörknöchelchen oder direkt an die Cochlea angekoppelt. Durch Anregung mit einer Wechselspannung werden elektrische Schallsignale in mechanische Schwingungen umgewandelt und an das Innenohr übertragen. In Deutschland besteht eine Zulassung zur Hörrehabilitation von Patienten mit sensorineuraler, Schallleitungs- oder kombinierter Schwerhörigkeit. Eine Vielzahl von Studien belegt den Nutzen und die Zuverlässigkeit dieses AMEI und damit auch des FMT [1–4].

Zur Versorgung von hochgradiger Schwerhörigkeit und Taubheit stehen diverse teilimplantierbare Cochleaimplantatsysteme zur Verfügung. Diese Systeme erfordern den Gebrauch eines externen Sprachprozessors, der unter anderem das Mikrofon und die Energieversorgung umfasst. Vollimplantierbare Systeme würden einige Vorteile bieten. Beispielsweise würde die Möglichkeit, diese Systeme kontinuierlich zu tragen – auch nachts oder unter speziellen Bedingungen wie beim Tragen eines Helms – eine durchgehende auditive Unterstützung und damit eine verbesserte Lebensqualität für die Nutzerinnen und Nutzer bedeuten.

Es wurden bereits vollimplantierbare Hörsysteme entwickelt, sie finden aber aus verschiedenen Gründen keine Anwendung [5–7]. Während auf dem Gebiet der Akkumulatoren durch die Entwicklungen der letzten Jahre realistische Lösungen für eine implantierbare, zuverlässige, transkutan wieder aufladbare Energiequelle vorliegen [8], stellt das für die Schallaufnahme zwingend notwendige implantierbare Mikrofon weiterhin eine Herausforderung dar. Ein implantierbares Mikrofon muss biokompatibel und hermetisch dicht sein. Größe und Form müssen eine sinnvolle otochirurgische Platzierung erlauben, und darüber hinaus muss sich die Klangqualität mit der von externen Mikrofonen messen können [9].

Verschiedene Positionierungen von implantierbaren Mikrofonen sind beschrieben. So z. B. im Fall von bereits entwickelten Implantatsystemen im Mastoid (Esteem, Fa. Envoy, Saint Paul/MN, USA) [10], subkutan (Carina und TIKI, Fa. Cochlear, Sydney, Australien) [11] und in der hinteren Gehörgangswand (TICA, Fa. Implex, München, Deutschland) [7].

Im Rahmen von Studien sind auch Positionierungen von Mikrofonsystemen im Mittelohr und der Cochlea erforscht [12–16]. Die beschriebenen Entwicklungen nutzen dabei ein piezoelektrisches Prinzip oder das Prinzip eines Kondensatormikrofons zur Wandlung von Schall in ein elektrisches Signal. Der FMT der Vibrant Soundbridge® (VSB) ist zur Schallerzeugung und Schallabgabe konzipiert. Im FMT bewegt sich dazu eine Masse durch Induktion in einem elektromagnetischen Feld im Kern einer Spule. Da das Prinzip der Induktion auch invertiert genutzt werden kann, ähnelt dieser Aufbau dem eines Tauchspulenmikrofons. Somit kann die Funktionsweise des FMT umgekehrt werden und dieser zur Schallaufnahme verwendet werden. Die vorliegende Pilotstudie untersucht diese Möglichkeit einer inversen Nutzung des FMT und die Eigenschaften bei der Verwendung als Mikrofon im Sinne eines Pilotprojekts.

## Material und Methoden

**Abb. 1:**
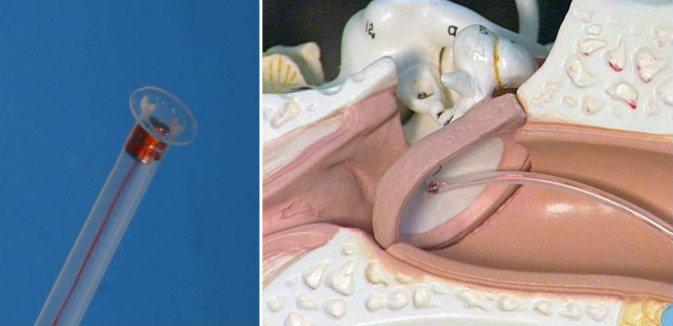
DDS-Testschwinger mit FMT im Silikonträger und Simulierung der Ankopplung an das Trommelfell im Modell (nicht maßstabsgetreu)

Die sog. Direct Drive Simulation (DDS) ist eine präoperative Klangsimulation vor der Implantation eines AMEI. Dabei wird ein in einem Silikonschlauch platzierter Direct Drive FMT (DD-FMT) an das Trommelfell oder die Paukenabdeckung des Patienten angekoppelt (Abb. 1). So können präoperativ realistische Klangsimulationen (Sprache und Musik) ermöglicht werden [17, 18]. In der vorliegenden Arbeit wurde der bei der DDS als Signalgeber genutzte DD-FMT in umgekehrter Weise, also als Schallaufnehmer (Mikrofon), getestet. Hierfür war dieser über einen vorgeschalteten rauscharmen Vorverstärker (LT1115, Fa. Linear Technology, Milpitas, USA) mit einer externen Soundkarte (Fireface UC, Fa. RME Intelligent Audio Solutions, Haimhausen, Deutschland) verbunden. Der DD-FMT entspricht in seinem Aufbau exakt dem FMT, der zur Implantation genutzt wird.

**Abb. 2:**
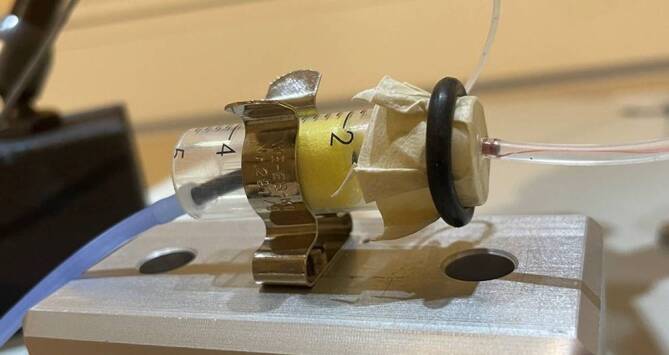
Trommelfell-Gehörgangs-Modell mit EAR3A, Referenzmikrofon und DD-FMT

Zur Untersuchung der Frequenzgänge des FMT als Mikrofon wurde ein vereinfachtes Trommelfell-Gehörgangs-Modell konstruiert (Abb. 2). Der Gehörgang wurde durch eine Polypropylen‑/Polyethylen-Spritze (Braun Injekt, Fa. Braun, Melsungen, Deutschland) nachgebildet. Als Gehörgangsvolumen wurden 2 ccm gewählt, entsprechend den gängigen Standards für Simulatoren des menschlichen Ohrs (DIN EN 60318-5:2007-04). An den Schaumstoffadapter wurde ein ER3A-Einsteckhörer (Fa. Etymotic, USA) als Schallgeber gekoppelt. Als Trommelfell diente ein Naturfell (Fa. Roland Meinl Musikinstrumente GmbH & Co. KG, Gutenstetten, Deutschland), welches mithilfe eines Gummirings gespannt wurde. Zur Referenzmessung des Schallpegels im Modell wurde ein kalibriertes Mikrofon (ER-7C, Fa. Etymotic, USA) über eine seitliche Bohrung vor das künstliche Trommelfell in das Gehörgangsmodell positioniert.

Als Testsignal zur Frequenzanalyse wurde ein linearer Chirp-Stimulus (100 Hz bis 10 kHz) dargeboten. Zur Bestimmung der Empfindlichkeit des FMT als Mikrofon wurde ein Sinuston der Frequenz 1 kHz mit 94 dB SPL genutzt.

Die Aufnahmen fanden in einer zertifizierten Audiometriekabine statt. Zur Signalaufnahme wurde der DD-FMT an die Membran des Trommelfell-Gehörgangs-Modells gekoppelt. Durch einen Tropfen Rizinusöl (Caesar & Loretz GmbH) war ein sicherer Halt auf der Membran gewährleistet (Abb. 2). Es war keine weitere Fixierung zwischen Membran und Fußplatte des DD-FMT notwendig.

Die Signale des Referenzmikrofons und des FMT wurden über die externe Soundkarte aufgenommen. Die Signalwandlung des analog eingehenden Signals fand durch den Analog-Digital-Wandler der Soundkarte statt. Der zeitliche Verlauf des digitalen Signals wurde über Matlab (The Math Works, Inc.) als Audiodatei im wav-Format mit 24 Bit und 48 kHz aufgezeichnet.

## Ergebnisse

Der Sitz des Einsteckhörers im künstlichen Gehörgang und die Ankopplung des DD-FMT an die Trommelfellnachbildung waren stabil. Dislokationen traten während der gesamten Messungen nicht auf (Abb. 2). Alle Hardware- und Softwarekomponenten des Aufbaus funktionierten fehlerfrei.

**Abb. 3:**
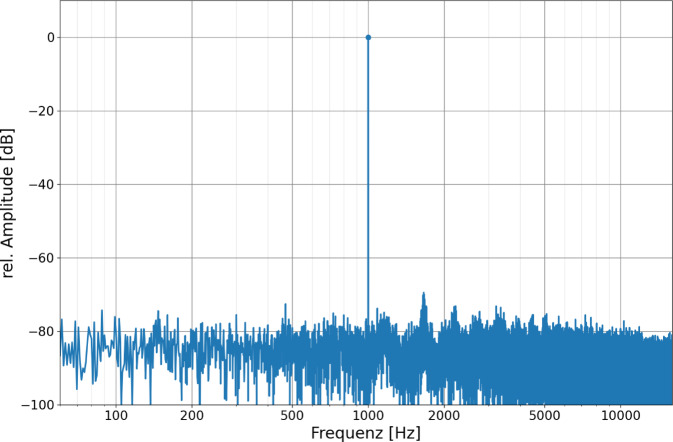
Frequenzgang des FMT bei Darbietung eines 1‑kHz-Sinustons mit 94 dB SPL

**Abb. 4:**
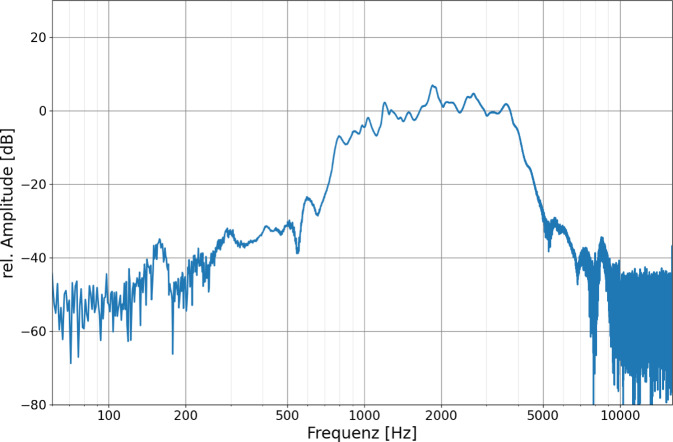
Frequenzgang des FMT bei Darbietung eines linearen Chirps (0,1–10 kHz)

Die Empfindlichkeit des FMT-Mikrofons wurde mit einem 1‑kHz-Sinuston mit einer Lautstärke von 94 dB SPL bestimmt. Hierbei konnte eine Ausgangsspannung von 51 µVeff, entspricht −26 dB (re 1 mPa) und ein Signal-Rausch-Abstand (SRA) von 79,3 dB ermittelt werden. Zur Darstellung der Empfindlichkeit und des Signal-Rausch-Abstands wurden die Amplituden auf 0 dB normiert (Abb. 3). Zudem wurde ein linearer Chirp-Stimulus im Frequenzbereich 0,1–10 kHz über den Einsteckhörer dargeboten, um den Frequenzgang des FMT zu beschreiben. Der Frequenzgang war nichtlinear. Die höchste Empfindlichkeit zeigte sich zwischen 1,5 und 2 kHz (Abb. 4). Die Amplituden nahmen mit niedrigeren und höheren Frequenzen ab.

## Diskussion

Vollständig implantierbare Hörsysteme würden für Patientinnen und Patienten einen erheblichen Mehrwert in Bezug auf die Lebensqualität bedeuten. So würde eine nahezu ununterbrochene Nutzungsmöglichkeit, abgesehen von Ladeintervallen, bestehen, z. B. auch in Situationen mit Wasserexposition wie beim Schwimmen oder Duschen. Zudem würde sich das Problem der sozialen Stigmatisierung lösen. Entscheidend für die Entwicklung solcher Systeme ist ein implantierbares Mikrofon, dessen technische Kennwerte, einschließlich Frequenzgang und Empfindlichkeit, vergleichbar sind mit aktuell in extern getragenen Sprachprozessoren verbauten Mikrofonen. Das Mittelohr erscheint nach unserer Einschätzung als der optimale Ort für die Platzierung eines derartigen Mikrofons.

Trotz jahrelanger Forschung verschiedener Arbeitsgruppen [11–16, 19] konnte bisher kein serientaugliches implantierbares Mittelohrmikrofon für die apparative Hörrehabilitation entwickelt werden. Aus Sicht der Hörrehabilitation hätte insbesondere ein im Mittelohr zu positionierendes Mikrofon interessantes akustisches Potenzial. So könnte ein solches System die sich aus der Form der Ohrmuschel und dem Gehörgang ergebenden natürlichen Frequenzgang und Laufzeitunterschiede des Schalls miterfassen. Somit stünden den Patient*innen zusätzliche Informationen zur Verfügung, die mit externen Mikrofonen nicht aufgenommen werden. Nachteile ergeben sich, wenn ein solches System Störungen aufweist, die nur durch eine Operation behoben werden können.

Zwar bestehen oder bestanden bereits Zulassungen für vollimplantierbare Systeme [5–7], diese nutzen aber zur Schallaufnahme keine im Mittelohr positionierten Mikrofone. In einer Reihe von Veröffentlichungen sind Modelle für Mittelohrmikrofone beschrieben, die sich entweder piezoelektrischer oder Kondensatorprinzipien bedienen [12, 14, 20]. Die vorliegende Arbeit untersucht als Pilotversuch zum ersten Mal die Mikrofontauglichkeit eines Masseschwingers, der eigentlich als Aktuator arbeitet. Dieser Masseschwinger befindet sich bereits im breiten klinischen Einsatz als Schallgeber in Form des FMT des teilimplantierbaren Mittelohrimplantats Vibrant Soundbridge®. Da sein Aufbau dem eines Tauchspulenmikrofons ähnelt, wurde in der vorliegenden Arbeit seine prinzipielle Verwendungsmöglichkeit als schallaufnehmender Wandler untersucht.

Die Untersuchungen zeigten einen nichtlinearen Frequenzverlauf des FMT. Die größte Empfindlichkeit lag zwischen 1500 und 2000 Hz. Hier zeigte sich der höchste Signal-Rausch-Abstand. Oberhalb und unterhalb dieses Bereichs nahm die Empfindlichkeit deutlich ab. Der für die Sprachverarbeitung noch nutzbare Frequenzbereich über dem Rauschen liegt bei dem untersuchten FMT etwa zwischen 500 Hz und 6 kHz.

Die Empfindlichkeit des FMT in dieser Studie ist mit −26 dB rms ref 1 mV gering. Eine Untersuchung eines piezoelektrischen implantierbaren Mikrofons zeigte eine mittlere Empfindlichkeit bei 1000 Hz von −44,22 dB rms ref 1 V bei Ankopplung an den Incus und −53,33 dB rms ref 1 V am Malleus [20].

Gérard et al. [21] untersuchten die Eignung eines implantierten Mikrofons für Cochleaimplantate anhand eines Vergleichs der Hörergebnisse mit externen Standardmikrofonen anhand von Reintonhörschwellen. Die Schwellen lagen in dieser Studie für das implantierte Mikrofon mit 44,9 dB höher als für das externe Mikrofon (36,4 dB). Angaben zur Empfindlichkeit der Mikrofonsysteme in dB rms ref 1 V finden sich jedoch nicht. Für Hörschwellenmessungen mit dem DD-FMT als Mikrofon sind weitere Untersuchungen mit Aufzeichnungen von Sinustönen in den gängigen Frequenzen der Reintonaudiometrie mit anschließender Darbietung an Cochleaimplantat(CI)-Träger über eine Direkteinkopplung unter Umgehung des eingebauten Mikrofons (z. B. via Bluetooth-Verbindung) geplant.

Es bestehen Einschränkungen in der vorliegenden Studie. Trotz sorgfältig ausgewählter Materialien muss davon ausgegangen werden, dass das Trommelfell-Gehörgangs-Modell in seinen Schwingungseigenschaften nicht dem natürlichen Schwingungsverhalten des Gehörgangs und des Trommelfells entspricht. Dieser Anspruch wurde für die erste Pilotstudie nicht gestellt.

Die gemessenen Werte sind daher stets unter Berücksichtigung dieses Fakts zu interpretieren und nicht als reale Empfindlichkeits- und Frequenzwerte zu betrachten.

## Fazit für die Praxis

- Die vorliegende Studie zeigt, dass sich der FMT grundsätzlich als Mikrofon nutzen lässt.
- Am Trommelfell-Gehörgangs-Modell hatte er eine nichtlineare Aufnahmecharakteristik und eine geringe Empfindlichkeit.
- Im sprachrelevanten Frequenzbereich zwischen 0,5 und 6 kHz lag in diesem vereinfachten Versuchsaufbau ein vielversprechender Signal-Rausch-Abstand (SRA) vor.
- Gelänge es, in einem Implantat die geringe Empfindlichkeit und die Nichtlinearität durch eine entsprechende Klangbearbeitung auszugleichen, bestünde die interessante Möglichkeit des Einsatzes als Mittelohrmikrofon.
- Die Studie ist ermutigend zur Weiterentwicklung eines leistungsstärkeren FMT mit einer höheren Empfindlichkeit und zur Durchführung weiterführender Untersuchungen am Felsenbeinmodell zur genaueren Bestimmung des Frequenzgangs, der Empfindlichkeit und des Grundrauschens.

## Data Availability

Die erhobenen Datensätze können auf begründete Anfrage in anonymisierter Form beim korrespondierenden Autor angefordert werden. Die Daten befinden sich auf einem Datenspeicher am Rechenzentrum der Klinik und Poliklinik für Hals-Nasen-Ohrenheilkunde, Kopf- und Hals-Chirurgie des Universitätsklinikums Würzburg.
